# Effect of Fluorescent Adhesives on Visibility and Tooth Enamel Surface Properties After Debonding: A Systematic Review and Meta‐Analysis

**DOI:** 10.1002/cre2.70385

**Published:** 2026-06-07

**Authors:** Maryam Omidkhoda, Erfan Bardideh, Alireza Nowabadi, Mostafa Entezari

**Affiliations:** ^1^ Orthodontic Department, Dental School Mashhad University of Medical Sciences Mashhad Iran; ^2^ Private Practice Mashhad Iran; ^3^ Mashhad University of Medical Sciences Mashhad Iran

**Keywords:** dental bonding, dental enamel, fluorescence, orthodontic brackets

## Abstract

**Objectives:**

To systematically review and meta‐analyze the impact of fluorescent versus conventional orthodontic adhesives on debonding efficiency, bond strength, and enamel integrity.

**Methods:**

This systematic review and meta‐analysis, following the PRISMA guidelines, searched PubMed, Scopus, Embase, Web of Science, and Cochrane Central (inception to November 2024) for studies comparing fluorescent versus conventional adhesives. Included were 19 studies (17 in vitro and 2 in vivo), with 11 meta‐analyses for shear bond strength (SBS), adhesive remnant index (ARI), residual adhesive surface area, enamel damage index (EDI), and removal time using a random‐effects model. The risk of bias was assessed with CRIS, RoB 2, and ROBINS‐I.

**Results:**

Of 798 records, 19 were included. Fluorescent adhesives reduced residual adhesive area (MD −0.57 mm^2^, 95% CI: −1.13, −0.02, *p* = 0.04) and removal time (MD −24.89 s, 95% CI: −50.29, 0.51, *p* = 0.05), but showed no significant differences in SBS (MD 1.07 MPa, 95% CI: −3.05, 5.19, *p* = 0.61), ARI (MD 0.18, 95% CI: −0.10, 0.46, *p* = 0.20), or EDI (MD −0.04, 95% CI: −0.28, 0.20, *p* = 0.73). Heterogeneity was high (e.g., *I* ^2^ = 99.77% for removal time), and most studies had a moderate bias. Narratively, fluorescence improved remnant detection (e.g., 50 µm via QLF).

**Conclusion:**

Fluorescent adhesives enhance debonding efficiency by reducing residual adhesive area and removal time in laboratory settings, without affecting bond strength. Enamel protection is operator‐dependent. Due to the predominance of in vitro studies (17/19) and limited clinical data (only 2 in vivo studies, one with a single patient), firm clinical recommendations cannot be made. High heterogeneity and moderate risk of bias further limit the strength of the findings. Further standardized clinical trials are needed.

## 1 Introduction

Orthodontic treatment relies heavily on adhesive systems to bond brackets to enamel surfaces, ensuring stability throughout the therapy (Mandall et al. 2018). Post‐treatment, complete adhesive removal is crucial to restore enamel integrity and prevent plaque accumulation; however, this process risks enamel damage if remnants persist (Hosein et al. 2004). Conventional adhesives, while effective, often leave residues challenging to detect under standard lighting, complicating cleanup and increasing procedure time (Grazioli et al. 2021). Fluorescent orthodontic adhesives incorporating UV‐sensitive or photochromic agents have emerged as a promising innovation to enhance visibility during debonding, potentially improving efficiency and reducing enamel harm (Namura et al. 2010; Ribeiro et al. 2017). Studies suggest that these adhesives may maintain adequate shear bond strength (SBS) while aiding remnant identification, although their impact on enamel surface properties remains debatable (Rossato et al. 2020; Farronato et al. 2021).

Despite growing interest, evidence is fragmented with in vitro and limited in vivo studies reporting variable outcomes of SBS, adhesive remnant index (ARI), enamel damage, and removal time (AlSamak et al. 2023; Stadler et al. 2019). Systematic reviews have addressed the conventional adhesive performance (Mandall et al. 2018), but none have comprehensively evaluated fluorescent adhesives across these parameters. This systematic review and meta‐analysis aims to synthesize data from 19 studies (2010–2024) to assess the efficacy of fluorescent versus conventional adhesives on visibility during debonding as well as enamel surface properties post‐debonding, focusing on adhesive remnant detection (visibility), SBS, ARI, residual adhesive surface area, enamel damage index (EDI), and removal time, to inform clinical practice and future research.

## 2 Methods and Materials

This systematic review and meta‐analysis were conducted following the Preferred Reporting Items for Systematic Reviews and Meta‐Analyses (PRISMA) guidelines (Moher et al. 2009) and adhered to a pre‐registered protocol on PROSPERO (registration number: CRD420251064518). The study was conducted at the School of Dentistry, Mashhad University of Medical Sciences, with a literature search spanning October 26, 2024, to November 4, 2024. This study was approved by the Ethics Committee of the School of Dentistry on March 17, 2024 (approval code: IR.MUMS.DENTISTRY.REC.1403.041). The objective of this study was to evaluate the effects of fluorescent adhesives on enamel surface properties after orthodontic debonding compared to conventional adhesives, focusing on the SBS, ARI, residual adhesive surface area, EDI, enamel loss, surface roughness, and adhesive removal time.

### 2.1 Eligibility Criteria

Studies were selected based on the following Population/Problem, Intervention, Comparison, and Outcome (PICO) framework:

#### 2.1.1 Population/Problem

Teeth subjected to orthodontic bonding (in vitro or in vivo).

#### 2.1.2 Intervention

Orthodontic adhesives containing fluorescent materials.

#### 2.1.3 Comparison

Conventional adhesives without fluorescent materials.

#### 2.1.4 Outcome

Differences in enamel loss, SBS, ARI, surface roughness, residual adhesive surface area, and adhesive removal time.

The inclusion criteria were as follows: (1) laboratory and clinical studies examining the effect of fluorescent adhesives on enamel surface properties post‐debonding and (2) studies reporting quantitative data on at least one specified outcome. Exclusion criteria included: (1) studies unrelated to dentistry; (2) non‐orthodontic studies (e.g., restorative dentistry, oral and maxillofacial surgery); (3) systematic reviews; (4) case reports; (5) studies focused solely on white spot lesions or demineralization unrelated to adhesive removal; (6) studies on splint trauma; and (7) studies involving clear aligner attachments without bracket debonding.

Although fluorescent adhesives are primarily designed for improved visibility, studies were included if they reported any quantitative data on enamel surface properties or visibility‐related outcomes after debonding, as these represent the core clinical concerns surrounding their use. We acknowledge that results (particularly for bond strength) vary depending on experimental conditions and materials used.

### 2.2 Search Strategy

A systematic literature search was conducted across PubMed, Scopus, Embase, Web of Science, and Cochrane Central Register of Controlled Trials from their inception to November 4, 2024, without restrictions on the language or publication date. The search strategies combined keywords and MeSH terms tailored to each database (Table 1). For example, the PubMed search used: (“Composite” OR “bond” OR “adhesive‐remnant index” OR “ARI” OR “glass Ionomer” OR “RMGI” OR “shear bond strength” OR “roughness” OR “enamel loss” OR “retainer” OR “retention” OR “Identification Technique” OR “FIT”) AND (“orthodontics”[MeSH Terms] OR “bracket” OR “fixed appliance” OR “self*ligate” OR “orthodontic”) AND (“fluoresce*” OR “Fluorophores” OR “ultraviolet” OR “UV”). Non‐English titles and abstracts were translated by a specialized individual. The reference lists of the included studies were manually searched for additional relevant articles.

**Table 1:** Systematic search strategies used for databases.

Databases. Applied search strategy and numbers of retrieved studies		
Database of published trials, dissertations and conference proceedings	Search strategy used	Hits
MEDLINE searched via PubMed searched on October 27, 2024, via www.ncbi.nlm.nih.gov/sites	(Composite OR bond OR adhesive‐remnant index OR ARI OR glass Ionomer OR RMGI OR shear bond strength OR roughness OR enamel loss OR retainer OR retention OR Identification Technique OR FIT) AND (“orthodontics”[MeSH Terms] OR bracket OR fixed appliance OR self*ligate OR orthodontic) AND (fluoresce* OR Fluorophores OR ultraviolet OR UV)	279
Web of Science Core Collection was searched via web of knowledge on October 27, 2024, via apps.webofknowledge.com	TS = (composite OR bond OR “adhesive‐remnant index” OR ari OR “glass Ionomer” OR rmgi OR “shear bond strength” OR roughness OR “enamel loss” OR retainer OR retention OR “Identification Technique” OR fit) AND TS = (orthodontic OR bracket OR “fixed appliance” OR “self ligate”) AND TS = (fluoresce* OR ultraviolet OR uv)	169
EMBASE searched via Ovid on October 26, 2024, via http://ovidsp.dc2.ovid.com	#1 ‘composite’/exp OR ‘composite’ OR ‘bond’/exp OR ‘bond’ OR ‘adhesive remnant index’/exp OR ‘adhesive remnant index’ OR ‘glass ionomer’/exp OR ‘glass ionomer’ OR ‘ari’ OR rmgi OR sbs OR ‘shear bond strength’/exp OR ‘shear bond strength’ OR ‘enamel loss’	108
	#2 ‘orthodontics’ OR ‘brackets’ OR ‘fixed appliance’	
	#3 fluoresce* OR ‘fluorescence’/exp OR ‘fluorescence’ OR ‘ultraviolet radiation’/exp OR ‘ultraviolet radiation’ OR ‘uv’/exp OR uv	
	#1 AND #2 AND #3	
Scopus searched via Scopus on October 28, 2024, via https://www.scopus.com	TITLE‐ABS‐KEY (composite OR bond OR “adhesive‐remnant index” OR ari OR “glass Ionomer” OR rmgi OR “shear bond strength” OR roughness OR “enamel loss” OR retainer OR retention OR “Identification Technique” OR fit) AND TITLE‐ABS‐KEY (orthodontic OR bracket OR “fixed appliance” OR “self ligate”) AND TITLE‐ABS‐KEY (fluoresce* OR ultraviolet OR uv)	192
Cochrane Central Register of Controlled Trials searched via the Cochrane Library Searched on October 27, 2024, via www.thecochranelibrary.com	#1 composite OR bond OR “adhesive‐remnant index” OR ari OR “glass Ionomer” OR rmgi OR “shear bond strength” OR roughness OR “enamel loss” OR retainer OR retention OR “Identification Technique” OR fit	50
	#2 MeSH descriptor: [Orthodontics] explode all trees	
	#3 orthodontic OR bracket OR “fixed appliance” OR “self ligate”	
	#4 fluoresce* OR ultraviolet OR uv	
	#5 #1 AND (#2 OR #3) AND #4	
Total		798

### 2.3 Study Selection

**Figure 1:**
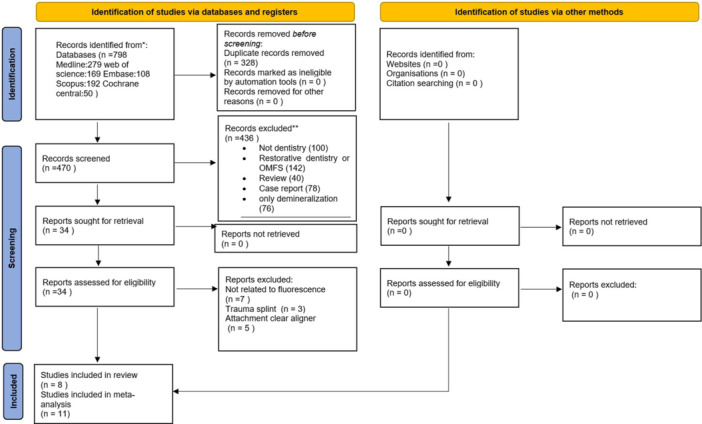
PRISMA flow diagram illustrating the study selection process.

A total of 798 records were identified through database searches (PubMed, 279; Web of Science, 169; Embase, 108; Scopus, 192; and Cochrane Central, 50). After removing 328 duplicates, titles and abstracts of 470 unique records were independently screened by three reviewers (M.O., A.N., and E.B.) against the eligibility criteria. Of these, 436 were excluded because they did not meet the inclusion criteria: 100 were unrelated to dentistry; 142 focused on restorative dentistry or oral and maxillofacial surgery; 78 were case reports; 40 were reviews; and 76 examined only demineralization. The full texts of the remaining 34 studies were retrieved and assessed, with 15 excluded (7 unrelated to fluorescence, 3 examining splint traumas, and 5 focusing on clear aligner attachments). Ultimately, 19 studies were included in the systematic review (Namura et al. 2010; Ribeiro et al. 2017; Rossato et al. 2020; Farronato et al. 2021; AlSamak et al. 2023; Stadler et al. 2019; Albertini et al. 2022; Schott and Meller 2018; Chung et al. 2024; Wu et al. 2023; Kim et al. 2018; Yamagata et al. 2018; Yan et al. 2024; Engeler et al. 2021; Moecke et al. 2022; Kaneshima et al. 2018; Magni et al. 2022; Lai et al. 2019; Bush et al. 2010; Rocha et al. 2017; Albertini et al. 2020; Ozcan et al. 2008), with 11 eligible for meta‐analysis and 8 included in narrative synthesis. The selection process was documented using the PRISMA flow diagram (Figure 1).

### 2.4 Data Extraction

Data were extracted by one reviewer (A.N.) and verified by another (E.B.) using predesigned forms. The extracted variables included (1) study characteristics (author, year, study type), (2) sample details (number and type of teeth in treatment and control groups), (3) adhesive details (type, concentration, and fluorescence vs. conventional), (4) outcome measures (SBS in MPa, ARI scores, residual adhesive surface area in mm^2^, surface roughness in Ra values, EDI, enamel loss in μm, removal time in seconds), and (5) study criteria (inclusion/exclusion details). Discrepancies were resolved through consensus.

### 2.5 Quality Assessment

The risk of bias for all 19 included studies was assessed by two reviewers (A.N. and E.B.). Laboratory studies were evaluated using the checklist for reporting in vitro studies (CRIS), assessing reproducibility, transparency, and assessability across domains, such as study design, controls, statistical methods, and results reporting. Studies were scored as high‐quality (80%–100%), moderate (50%–79%), or low (< 50%). Clinical randomized trials were assessed using Cochrane's Risk of Bias 2 (RoB 2) tool, covering five domains (randomization, deviations, missing data, outcome measurement, and result selection), with judgments of low risk, concern, or high risk. Non‐randomized clinical studies were evaluated using Cochrane's ROBINS‐I tool, addressing seven domains (confounding, selection, intervention classification, deviations, missing data, outcome measurement, and result selection) with judgments of low, moderate, serious, or critical risk. Results were summarized narratively and in tables.

### 2.6 Statistical Analysis

Meta‐analysis was restricted to 11 in vitro studies due to high variability in clinical study outcomes using Stata software version 18 (StataCorp, College Station, TX, USA). A random‐effects inverse variance model with Restricted Maximum Likelihood was applied to calculate mean differences (MD) with 95% confidence intervals (CIs). Heterogeneity was assessed using the *τ* ^2^ and *I* ^2^ values, with *I* ^2^ > 50% indicating substantial heterogeneity. Pre‐planned subgroup analyses by dental sample type (bovine vs. human teeth) were conducted when data permitted. Due to the small number of studies per outcome and confounding between adhesive type and illumination method, formal subgroup meta‐analysis by illumination type (UV/FIT vs. conventional) was not feasible. Heterogeneity sources were further explored through sensitivity analyses and narrative synthesis.

## 3 Results

### 3.1 Study Selection

A total of 798 records were retrieved from the database searches (PubMed, 279; Web of Science, 169; Embase, 108; Scopus, 192; Cochrane Central, 50). After removing 328 duplicates, 470 unique records were screened for title and abstract. Of these, 436 were excluded because they did not meet the eligibility criteria: 100 were unrelated to dentistry; 142 focused on restorative dentistry or oral/maxillofacial surgery; 78 were case reports; 40 were reviews; and 76 examined only demineralization. The full texts of 34 studies were assessed, with 15 excluded (7 unrelated to fluorescence, 3 on splint trauma, and 5 on clear aligner attachments). Ultimately, 19 studies met the inclusion criteria (Namura et al. 2010; Ribeiro et al. 2017; Rossato et al. 2020; Farronato et al. 2021; AlSamak et al. 2023; Stadler et al. 2019; Albertini et al. 2022; Schott and Meller 2018; Chung et al. 2024; Wu et al. 2023; Kim et al. 2018; Yamagata et al. 2018; Yan et al. 2024; Engeler et al. 2021; Moecke et al. 2022; Kaneshima et al. 2018; Magni et al. 2022; Lai et al. 2019; Bush et al. 2010; Rocha et al. 2017; Albertini et al. 2020; Ozcan et al. 2008). Eleven studies were included in meta‐analysis, while eight were narratively synthesized due to heterogeneity. The selection process is detailed in the PRISMA flow diagram (Figure 1).

### 3.2 Characteristics of Included Studies

The 19 included studies, published between 2010 and 2024, comprised 17 in vitro studies and 2 in vivo studies, with one study (Rossato et al. 2020) including both in vitro and clinical phases. The 11 studies in the meta‐analysis focused on the SBS, ARI, residual adhesive surface area, EDI, and adhesive removal time. The remaining eight studies reported additional outcomes (e.g., enamel damage volume/depth and fluorescence intensity) unsuitable for meta‐analysis. The study characteristics are summarized in Table 2.

**Table 2:** Characteristics and extracted data of included studies.

No.	Study (Author/Year)	Study design and sample	Adhesive types/groups	Key variables/outcomes	Key results/conclusions
1	Ribeiro et al. (2017)	In vitro 38 premolars (19 conventional lighting vs. 19 UV lighting) *Brackets:* Standard orthodontic metal brackets	‒ Fluorescent adhesives (Opal Bond MV) + UV flashlight vs. fluorescent adhesives (Opal Bond MV) + conventional light	‒ Adhesive remnant index (ARI) (area in mm^2^) ‒ Enamel damage index (EDI) ‒ SEM observation	‒ UV group had significantly lower adhesive remnants (median ARI: 0.25 vs. 0.80 mm^2^; *p* < 0.001) ‒ No difference in enamel damage (*p* = 0.729) ‒ Concluded UV light improves visualization and removal efficiency without additional enamel harm
2	Rocha et al. (2017)	In vitro 40 bovine enamel surfaces, 4 groups (C, W, F, FL) *Brackets:* Not specified	‒ C: Conv. handpiece ‒ W: Handpiece + white LED ‒ F: Handpiece + fluor LED ‒ FL: Curing light + fluor lens (for bracket removal) ‒ All group Opal Bond MV (fluorescent)	‒ Enamel thickness (pre vs. post) ‒ Surface resin residue ‒ Adhesive tag depth ‒ Enamel wear	‒ FL showed highest enamel wear but lowest resin residue ‒ Groups C and W had largest remaining resin ‒ F and FL had more enamel wear (complete resin removal) ‒ Tag depth ~8.7 μm; influenced by viscosity and acid etching
3	Farronato et al. (2021)	In vitro, blind randomized 48 extracted human teeth (24 fluorescent composite vs. 24 standard) *Brackets:* Leone stainless steel brackets	‒ DFC: BrackFix (Voco) fluorescent composite ‒ DSC: Transbond XT (3 M) conventional non‐fluorescent	‒ Modified ARI (5× magnification) ‒ Duration of bracket de‐bonding (s)	‒ Fluorescent composite (DFC) led to lower ARI and shorter removal time (38 s vs. 77 s) ‒ Minimal enamel damage with DFC
4	Engeler et al. (2021)	In vitro 112 tooth surfaces (buccal and lingual) FIT groups vs. non‐FIT *Brackets:* Lingual (custom Incognito) and buccal (standard)	‒ FIT adhesives (OPAL, BRACE) vs. Conventional adhesive (Trans bond XT) ‒ Lighting or method specifics not fully detailed	‒ Composite remnant height/volume ‒ Enamel defect volume/depth ‒ Cleanup time	‒ FIT (OPAL, BRACE) achieved complete composite removal (buccal) but had larger enamel defect volume lingually ‒ FIT significantly reduced cleanup time on buccal surfaces; slightly slower on lingual ‒ Enhanced visualization and thorough cleanup
5	AlSamak et al. (2023)	In vitro 90 extracted maxillary 1st premolars (9 groups × 10 teeth) *Brackets:* Pinnacle System—Roth	9 adhesives tested:	‒ Shear bond strength (SBS) ‒ Adhesive remnant index (ARI)	‒ Color‐change and fluorescent adhesives generally higher SBS vs. conventional ‒ Lowest SBS: Transbond Plus + GoTo (TBPGT) ‒ No significant ARI differences overall; bond failures mostly cohesive in adhesive
			1. GoTo (Reliance)—FOA 2. Light bond medium (Reliance)—COA 3. Greengloo (Ormco)— CCOA 4. Enlight (Ormco)—COA 5. Transbond plus (3 M)—CCOA 6. Transbond XT (3 M)—COA 7. BracePaste color change (American Orth.)—CCOA 8. BracePaste adhesive (American Orth.)—COA 9. Transbond plus + GoTo (TBPGT) – Mixed color‐change + fluorescent		
6	Namura et al. (2010)	In vitro 64 bovine teeth/4 groups ×16 *Brackets:* Metal, 15.26 mm^2^ base	‒ F1/F2/F3: 0.001%, 0.002%, 0.003% coumarin‐derived fluorescent dye (Okayama) in adhesive ‒ Control: Transbond XT	‒ SBS (immediate, 24 h, thermal cycle) ‒ ARI ‒ Fluorescence intensity ‒ Color penetration through clear brackets	‒ Up to 0.002% dye = adequate SBS + good fluorescence ‒ 0.003% dye had strong fluorescence but significantly lower SBS ‒ F1 and F2 are clinically acceptable combos for visualization
7	Kaneshima et al. (2018)	In vitro 60 human molars, 3 adhesive groups (opaque, low fluor, high fluor) × 2 removal methods (no UV vs. UV)	‒ Opaque resin (Enlight) vs. low fluorescence (Transbond color change) vs. high fluorescence (Orthocem UV Trace) ‒ No UV vs. UV for removal	‒ Time to remove remnant ‒ Direct visual and SEM marks ‒ ARI	‒ UV‐aided removal was faster but yielded more evident marks ‒ Polishing reduced marks across all adhesives ‒ No major difference in ARI or enamel preservation among adhesives
8	Lai et al. (2019)	In vitro 80 premolars, 4 groups (20 each), comparing UV vs. white light	‒ Pad Lock vs. Opal Bond MV adhesives both fluorescent ‒ UV vs. white light	‒ Adhesive remnant surface area ‒ Removal time	‒ Under UV, significantly less remnant area and shorter removal for Opal Bond MV ‒ No difference in removal time for Pad Lock adhesives ‒ UV detection not reliable for sub‐2 µm thickness
9	Stadler et al. (2019)	In vitro 120 teeth (12 models; FIT vs. CLS groups) *Brackets:* Not specified	‒ Fluorescence‐aided technique (FIT) vs. Conventional light source (CLS)	‒ Composite remnant (height, volume) ‒ Enamel defect (depth, volume) ‒ Cleanup time	‒ FIT significantly reduced composite remnants and cleanup time ‒ Less enamel loss with fluorescent illumination ‒ Operator variability minimal
			Both Opal Bond		
10	Moecke et al. (2022)	In vitro 45 human upper central incisors (3 groups × 15) *Brackets:* Not specified	‒ BF/UV: Fluor + UV (BrackFix) ‒ BF/0: Fluor no UV (BrackFix) ‒ TB/0: Non‐fluor (Transbond XT)	‒ Adhesive remnant % (stereomicroscope) ‒ Removal time	‒ BF/UV group: lowest adhesive remnant and fastest removal ‒ BF/0 and TB/0 took longer and left more residue ‒ No enamel damage data presented
11	Yan et al. (2024)	In vitro 40 premolars (removal analysis), 105 (immediate SBS), 60 (long‐term SBS) *Brackets:* GAC metal (1st premolar)	‒ Control: RMGI ‒ PCA with photochromic dye (Green by Light) (1%–20%) ‒ Final group: PCA5% best ratio	‒ Shear bond strength ‒ Cytocompatibility ‒ ARI ‒ Enamel damage (index) ‒ Excess adhesive ‒ Removal time	‒ PCA1–5% comparable SBS to RMGI; higher than 10 + % ‒ PCA5% gave smaller leftover but took slightly more removal time ‒ No additional enamel damage from photochromic adhesives
12	Asokan et al. (2024)	In vitro 40 extracted human premolars (4 groups ×10) *Brackets:* Conventional metal (MBT)	‒ G1: Enlight (conventional) ‒ G2: Grengloo (color change) ‒ G3: Brace Paste (conventional) ‒ G4: Enli ght + UV dye	‒ SBS (Universal test machine) ‒ ARI (stereomicroscope) ‒ Enamel damage index (EDI) (SEM) ‒ Removal time ‒ Remnant surface area	‒ Grengloo = highest SBS; Enlight = lowest ‒ Brace Paste removed fastest; minimal enamel damage across all groups ‒ No significant difference in ARI or surface area
13	Rossato et al. (2020)	Two‐phase: In vitro + Clinical (split‐mouth) *In vitro:* 40 teeth; *Clinical:* 8 patients (160 teeth) *Brackets:* 3 M Oral Care tubes (lab), 3 M metal (clinical)	‒ Intervention (UV): Orthocem UV trace (FGM) ‒ Control: Transbond XT (3 M)	‒ In vitro: SBS, ARI ‒ Clinical: bond failure rate (2 years)	‒ In vitro SBS: no significant diff. (Orthocem UV = 12.69 MPa vs. Transbond=13.80 MPa) ‒ Clinical bond failure: 2.5% versus 5% (no sig. diff) ‒ Concluded fluorescent additive helps visualize remnants without harming bond strength
14	Chung et al. (2024)	In vitro: 5 orthodontic resins, 1 non‐fluorescent resin control (Transbond XT), 4 groups of disc‐shaped adhesive samples, 5 bovine teeth per group, QLF analysis, incubation in artificial saliva.	5 orthodontic resins (Opal Bond MV‐Brace Paste‐BrackFix‐Pad Lock‐GoTo), 1 non‐fluorescent resin control (Transbond XT).	QLF imaging (ΔR > 30%, simple hygiene score)	‒ GC Ortho and GOTO: higher fluorescence. ‒ Fluorescence increased with thickness. ‒ QLF detected adhesive residues and biofilm.
				‒ Adhesive thickness (0.1 mm, 0.3 mm, 0.5 mm, 0.8 mm)	
15	Wu et al. (2023)	In vitro: 12 bovine teeth, 10 isolated human premolar teeth, 4 adhesive types (GC Ortho, GOTO, T Orthobond, Transbond XT), 10 days incubation in artificial saliva.	GC Ortho, GOTO, T Orthobond, Transbond XT	‒ QLF imaging (ΔR > 30%, SHS) ‒ Adhesive thickness (0.1 mm–0.8 mm)	‒ GC Ortho and GOTO: higher fluorescence. ‒ Fluorescence increased with thickness. ‒ QLF detected adhesive remnants and biofilm accumulation.
16	Yamagata et al. (2018)	In vitro: 72 specimens, 8 versions of light‐curing resin blends with various concentrations of Eu(DBM)3Phen. Fabricated and polymerized in a stainless steel mold.	Dimethacrylate‐based resins doped with Eu(DBM)3Phen at different concentrations (0.1%, 0.2%, 0.4%)	‒ Transparency (Tt), haze ‒ Photoluminescence ‒ Emission intensity, CIE color difference	‒ Strongest luminescence at 0.1 wt% Eu(DBM)3Phen. ‒ Emission intensity plateaus at 0.2 wt%. ‒ Transparent with a pale yellow tint at higher concentrations. ‒ Promises effective adhesive removal post‐treatment.
17	Kim et al. (2018)	In vitro: 3 adhesive types (Transbond XT, Blugloo, Enlight). 30 human teeth (premolars and molars), adhesive discs prepared and ground to thicknesses ranging from 800 μm to 20 μm.	Transbond XT, Blugloo, Enlight	‒ QLF‐D imaging (fluorescence and white‐light) ‒ Color parameters (L*, a*, b* values) ‒ Thickness variations of residual adhesive	‒ Fluorescence images showed greater color difference (ΔEF) than white‐light (ΔEW). ‒ Fluorescence detected residual adhesive as thin as 50 μm, while white‐light images detected adhesive only at 400–800 μm thickness. ‒ Color difference increased with adhesive thickness, particularly in fluorescence images.
18	Albertini et al. (2022)	In vivo: 1 patient, 6 adhesives applied to upper anterior teeth.	Ortho Connect, Gradia LoFlo A3.5, Greengloo, Transbond XT, KommonBase Pink, KommonBase Clear.	‒ FIT fluorescence measurement ‒ Brightness difference (L* color coordinate)	‒ Ortho Connect, Gradia LoFlo, KommonBase Clear had highest fluorescence brightness. ‒ Viscosity did not affect fluorescence.
19	Schott and Meller (2018)	In vivo: Resin‐based bracket bonding remnants removed after debonding using fluorescence‐aided identification technique (FIT)	Transbond XT (conventional bonding) vs. BrackFix (fluorescent resin bonding)	‒ FIT light source for fluorescence (near UV light). ‒ Enamel preservation after debonding.	‒ FIT allowed easier and faster removal of fluorescent resin remnants. ‒ BrackFix illuminated under FIT showed clear distinction from tooth structure, minimizing enamel damage. ‒ Reduced time for adhesive removal.

### 3.3 Quality Assessment

**Figure 2:**
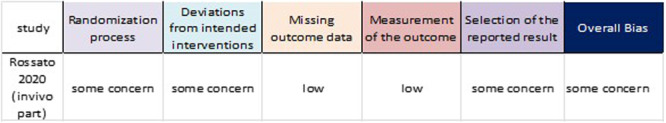
Risk of bias assessment of randomized studies using ROB 2.

**Figure 3:**
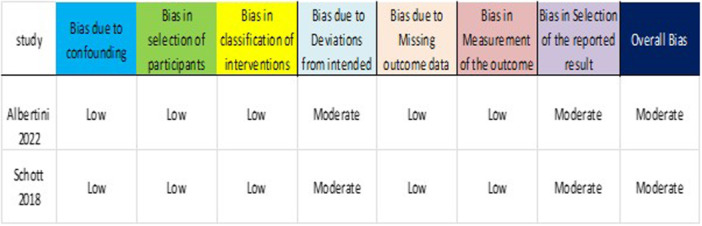
Risk of bias assessment of non‐randomized studies using ROBINS‐I.

The quality of the 17 in vitro studies was assessed using CRIS, evaluating randomization, blinding, standardization, and outcome reporting (Table 3). Most studies exhibited a moderate risk of bias, primarily owing to the lack of operator blinding (unavoidable with visible fluorescence) and unclear randomization. Farronato et al. (2021) achieved low risk due to blinded outcome assessment. The clinical phase of Rossato et al. (2020) was assessed using Cochrane's RoB 2, rated as “some concern” due to unclear allocation concealment and lack of registered protocols despite patient blinding (Figure 2). Previous in vivo studies (Albertini et al. 2022; Schott and Meller 2018) were evaluated using ROBINS‐I, both rated moderate risk due to no blinding and unregistered protocols, although there was a low risk for participant selection bias (Figure 3).

**Table 3:** Risk of bias assessment for studies included in the review.

No.	Study	Type of study	Sample and allocation	Blinding	Standardization	Outcome measures and reporting	Overall risk
**1**	Ribeiro et al. (2017)	In vitro	**Moderate**	**High**	**Moderate**	**Low**	**Moderate**
			38 premolars, 19 vs. 19. No explicit random method but equal group size.	Operator likely knew which group used UV flashlight. No mention of assessor blinding	Low‐speed bur used consistently, but operator variation possible	ARI and EDI measured with SEM and Mann–Whitney test. Clear stats	
**2**	Rocha et al. (2017)	In vitro	**Moderate**	**High**	**Moderate**	**Low**	**Moderate**
			40 bovine surfaces in 4 groups (C, W, F, FL); no randomization described.	Different lighting devices are obvious, no blinding of operator.	High‐speed handpiece technique described, some risk of operator subjectivity.	Data for resin residue, enamel wear, tag depth carefully reported, confocal imaging.	
**3**	Farronato et al. (2021)	In vitro	**Low**	**Low**	**Low**	**Low**	**Low**
			48 extracted teeth allocated (24 vs. 24) with stated “blind randomized design.”	The authors mention blinding the outcome assessor to composite type.	Standard bracket, same acid‐etch. Operator details well described.	ARI scoring and time to debond with robust comparisons.	
**4**	Engeler et al. (2021)	In vitro	**Moderate**	**High**	**Moderate**	**Low**	**Moderate**
			112 tooth surfaces, no explicit randomization.	Use of FIT vs. non‐FIT is likely not blinded.	Distinct groups (FIT adhesives vs. conventional) but operator skill unaccounted for.	Reporting of volumes, times, *p*‐values.	
**5**	AlSamak et al. (2023)	In vitro	**Moderate**	**Moderate**	**Low**	**Low**	**Moderate**
			90 premolars in 9 adhesive groups ×10 each. No mention of random assignment but balanced.	Operator cannot be blinded to brand or color‐change presence.	Standard SBS machine, ARI under stereomicroscope, widely accepted.	Comprehensive data on 9 adhesives, no sign of selective reporting.	
**6**	Namura et al. (2010)	In vitro	**Moderate**	**High**	**Moderate**	**Low**	**Moderate**
			64 bovine teeth in 4 groups. Not stated as random, but systematically allocated to dye concentrations.	Fluorescent dye levels differ obviously; no mention of blinding.	SBS tests, thermal cycling done, but no mention of multiple operators or calibration.	ARI, color difference, SBS all reported.	
**7**	Kaneshima et al. (2018)	In vitro	**Moderate**	**High**	**Moderate**	**Low**	**Moderate**
			60 molars, 3 adhesive groups, 2 removal methods. Balanced but no mention random.	UV vs. no‐UV is visible to operator.	Polishing steps described; potential operator variance.	Time to remove and SEM data well presented.	
**8**	Connie (2019)	In vitro	**Moderate**	**High**	**Moderate**	**Low**	**Moderate**
			80 teeth, 4 groups. Possibly convenience grouping.	Operator sees if they are using UV or white light, so no blinding.	Opal Bond vs. Pad Lock adhesives. Method is consistent but some potential variation.	Time and surface area measured by ImageJ, outcomes clearly reported.	
**9**	Stadler et al. (2019)	In vitro	**Low**	**High**	**Low**	**Low**	**Moderate**
			120 teeth systematically allocated to FIT vs. CLS. Not “random,” but large balanced groups.	Using a fluorescence‐aided technique is obvious, no blinding.	3D scanning, digital superimposition. Very standardized.	Comprehensive data, volumetric comparisons.	
**10**	Moecke et al. (2022)	In vitro	**Moderate**	**High**	**Moderate**	**Low**	**Moderate**
			45 teeth in 3 groups. No explicit randomization mentioned.	Knowing if UV is used is unavoidable.	Standard approach for removing adhesive, but skill differences possible.	Remnant % and removal times are straightforward.	
**11**	Yan et al. (2024)	In vitro	**Low**	**High**	**Moderate**	**Low**	**Moderate**
			Large sample: multiple sub‐experiments. Groups defined by photochromic ratio vs. control RMGI.	Dyes are visually distinct; no mention of blinding.	SBS and ARI standard, cytotoxic assays included. Good detail overall but operator aspects unclear.	Broad outcome reporting, including enamel damage index, ARI, time.	
**12**	Asokan et al. (2024)	In vitro	**Moderate**	**High**	**Low**	**Low**	**Moderate**
			40 premolars, 4 groups ×10. No randomization stated.	Different adhesives (Enlight, Grengloo, etc.) are recognizable.	SBS with universal machine, ARI with stereomicroscope, EDI with SEM.	All outcome data reported (SBS, ARI, EDI, removal time).	
**13**	Rossato et al. (2020) (Lab Part)	In vitro + Clinical	**Low**	**High**	**Moderate**	**Low**	**Moderate** (in vitro)
			40 teeth, 20 per group (UV vs. control). Possibly not random but well balanced.	Fluorescent vs. conventional known by operator.	SBS, ARI standard protocols, but no mention of repeated operator training.	Transparent results, stats for SBS and ARI.	
**14**	Chung et al. (2024)	In vitro	**Moderate**	**High**	**Low**	**Low**	**Moderate**
			Bovine teeth (12 per adhesive type) and isolated human premolars‐no mention of random allocation	No blinding mentioned in visual assessment	Standardized adhesive application and curing procedures, with consistent use of QLF for imaging and analysis.	Fluorescence intensity measured using QLF system, ΔR > 30%, and Simple Hygiene Score (SHS). Results clearly reported, with statistical analysis (*p*‐values).	
**15**	Wu et al. (2023)	In vitro	**Moderate**	**High**	**Low**	**Low**	**Moderate**
			Bovine teeth (12 per adhesive type) and isolated human premolars. No explicit mention of randomization.	No blinding mentioned. The operator could have been aware of the adhesive types used	Standardized adhesive application, curing procedures, and consistent use of QLF system for fluorescence imaging and analysis.	Fluorescence intensity, ΔR > 30%, and simple hygiene score (SHS) were obtained and analyzed using software. Statistical analysis included *p*‐values (ANOVA).	
**16**	Yamagata et al. (2018)	In vitro	**Moderate**	**High**	**Low**	**Low**	**Moderate**
			72 resin specimens made from Eu(DBM)3Phen doped composites. No mention of randomization in sample preparation.	No mention of blinding; potential detection bias due to fluorescence measurements.	Standardized procedure for resin preparation, light curing, and fluorescence measurements	Results clearly reported with CIE color differences (ΔE*ab).	
**17**	Kim et al. (2018)	In vitro	**Moderate**	**High**	**Low**	**Low**	**Moderate**
			30 human premolars and molars with adhesive discs. Specimens were not randomly allocated	No blinding mentioned could introduce potential bias	QLF‐D system was consistently used for fluorescence and white‐light imaging.	Fluorescence color (ΔE) and CIE Lab values* were used to compare residual adhesive thickness and color differences between adhesive and tooth	

### 3.4 Meta‐Analysis Results

Visibility‐related outcomes (residual adhesive surface area and adhesive removal time) were considered the primary outcomes most directly linked to the intended advantage of fluorescent adhesives (enhanced remnant detection under UV or fluorescence‐aided identification technique [FIT]). Meta‐analyses were conducted on 11 in vitro studies using a random‐effects model for five outcomes.

### 3.5 Shear Bond Strength (SBS)

**Figure 4:**
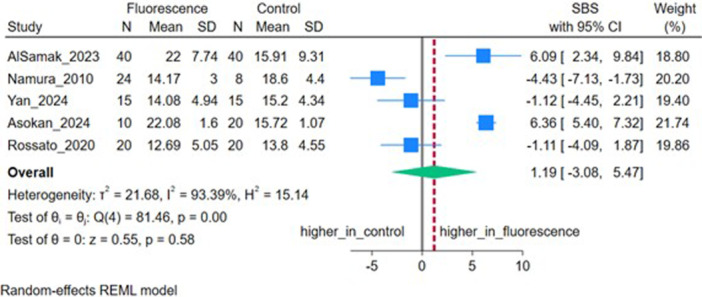
Forest plot depicting the shear bond strength (SBS) of included studies.

**Figure 5:**
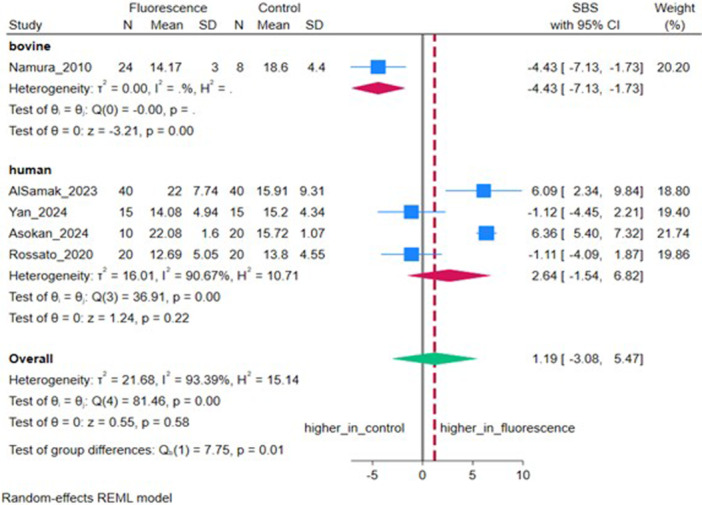
Forest plot depicting shear bond strength (SBS) stratified by tooth type.

Five studies (*n* = 5) reported SBS. The pooled MD was 1.07 MPa (95% CI: −3.05, 5.19), which was not statistically significant (*p* = 0.61), with high heterogeneity (*I* ^2^ = 93.07%, *τ* ^2^ = 20.01) (Figure 4). Sensitivity analysis excluding bovine samples showed no significant difference (*p* = 0.22) (Figure 5).

### 3.6 Adhesive Remnant Index (ARI)

**Figure 6:**
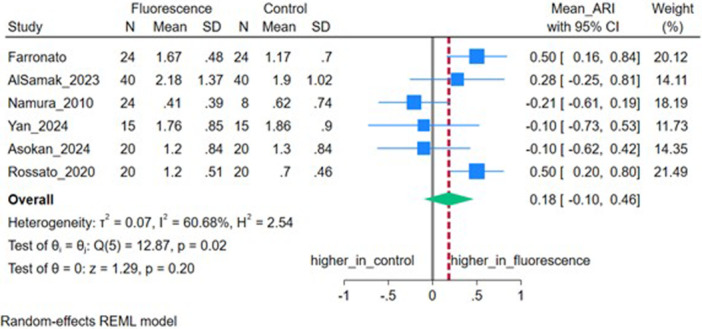
Forest plot depicting the adhesive remnant index (ARI) of included studies.

**Figure 7:**
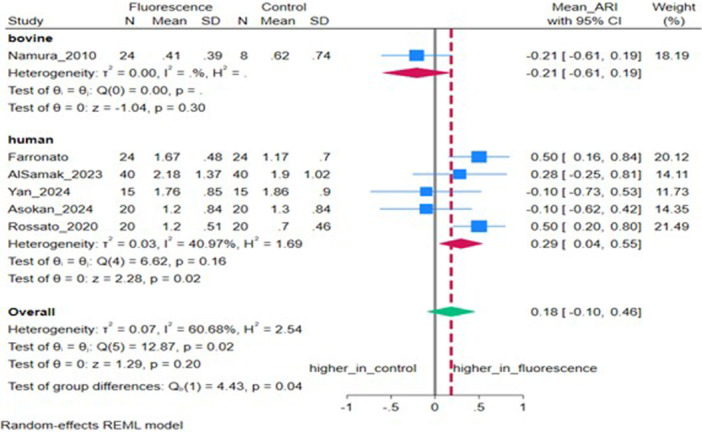
Forest plot depicting the adhesive remnant index (ARI) stratified by tooth type.

Six studies (*n* = 6) reported ARI. The pooled MD was 0.18 (95% CI: −0.10, 0.46), not significant (*p* = 0.20), with moderate‐to‐high heterogeneity (*I* ^2^ = 60.68%, *τ* ^2^ = 0.07) (Figure 6). Sensitivity analysis excluding bovine samples showed a significant increase in the ARI for fluorescent adhesives (*p* = 0.02) (Figure 7).

### 3.7 Residual Adhesive Surface Area

**Figure 8:**
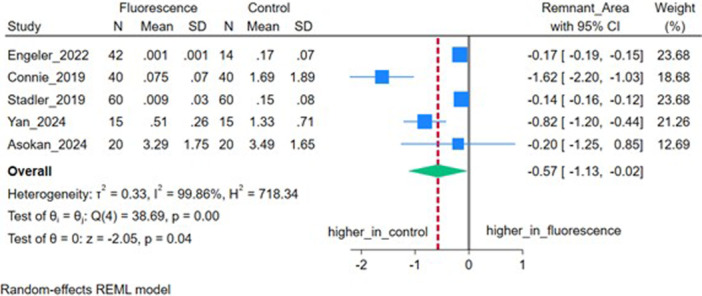
Forest plot depicting the extent of adhesive residue on tooth surfaces.

Five studies (*n* = 5) reported the residual adhesive surface area (a quantitative measure of remaining composite after cleanup that serves as an objective proxy for visibility‐assisted cleanup efficiency). The pooled MD was −0.57 mm^2^ (95% CI: −1.13, −0.02), which was significantly lower in the fluorescent group (*p* = 0.04), with very high heterogeneity (*I* ^2^ = 99.86%, *τ* ^2^ = 0.33) (Figure 8).

The significant reduction in residual adhesive surface area supports improved visibility and more complete cleanup when fluorescent adhesives are used with appropriate illumination.

### 3.8 Enamel Damage Index (EDI)

**Figure 9:**
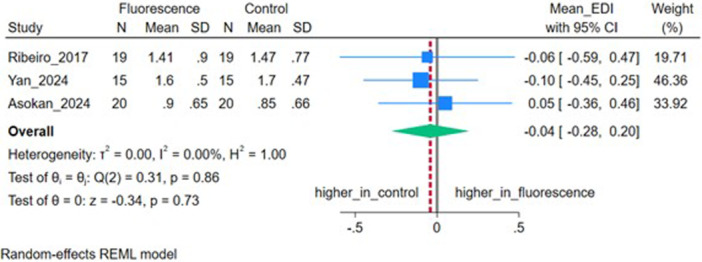
Forest plot depicting the enamel damage index (EDI) of included studies.

Three studies (*n* = 3) reported the EDI. The pooled MD was −0.04 (95% CI: −0.28, 0.20), not significant (*p* = 0.73), with no heterogeneity (*I* ^2^ = 0%, *τ* ^2^ = 0.00) (Figure 9).

### 3.9 Adhesive Removal Time

**Figure 10:**
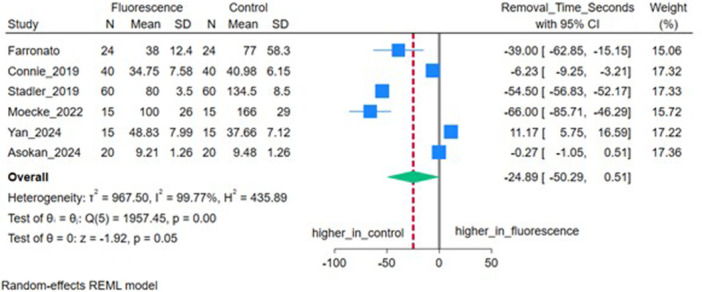
Forest plot depicting the duration required for adhesive removal.

Six studies (*n* = 6) reported the removal time. The pooled MD was −24.89 s (95% CI: −50.29, 0.51), borderline significant favoring fluorescent adhesives (*p* = 0.05), with very high heterogeneity (*I* ^2^ = 99.77%, *τ* ^2^ = 967.50) (Figure 10).

### 3.10 Narrative Synthesis

Eight studies were narratively synthesized due to heterogeneous outcome measures, including fluorescence intensity, enamel damage, and detection methodologies. These studies consistently demonstrated that fluorescence enhances visualization of adhesive remnants and facilitates more efficient removal under ultraviolet or FIT (Chung et al. 2024; Wu et al. 2023; Kim et al. 2018; Yamagata et al. 2018; Yan et al. 2024; Engeler et al. 2021; Moecke et al. 2022; Kaneshima et al. 2018; Magni et al. 2022; Lai et al. 2019; Bush et al. 2010; Rocha et al. 2017; Albertini et al. 2020; Ozcan et al. 2008; Asokan et al. 2024; Reynolds 1975).

Chung et al. (2024), Wu et al. (2023), and Kim et al. (2018) showed that fluorescence intensity increases with adhesive thickness, improving detection sensitivity. Yamagata et al. (2018) reported optimal luminescence at specific concentrations, while Yan et al. (2024) demonstrated that photochromic adhesives can maintain bond strength while reducing adhesive remnants.

In vivo investigations further supported the clinical applicability of fluorescence‐aided techniques (Albertini et al. 2022; Schott and Meller 2018), while Rossato et al. (2020) reported no difference in clinical bond strength.

### 3.11 Certainty of Evidence (GRADE)

The certainty of evidence for each outcome was assessed using the Grading of Recommendations Assessment, Development and Evaluation (GRADE) approach. All outcomes were downgraded due to serious risk of bias (predominantly moderate risk in in vitro studies using CRIS), very serious inconsistency (high heterogeneity), serious indirectness (predominantly in vitro evidence), and imprecision (small number of studies and wide confidence intervals in several outcomes). No serious concerns regarding publication bias were detected due to the limited number of studies per outcome. Overall, the certainty of evidence for all meta‐analyzed outcomes was rated as very low (Table 4).

**Table 4:** GRADE summary of findings.

Outcome	No. of studies	Risk of bias	Inconsistency	Indirectness	Imprecision	Publication bias	Certainty of evidence (GRADE)
Shear bond strength	5	Serious	Very serious	Serious	Serious	Undetected	Very low
Adhesive remnant index (ARI)	6	Serious	Serious	Serious	Serious	Undetected	Very low
Residual adhesive surface area	5	Serious	Very serious	Serious	Serious	Undetected	Very low
Enamel damage index (EDI)	3	Serious	Not serious	Serious	Very serious	Undetected	Very low
Adhesive removal time	6	Serious	Very serious	Serious	Serious	Undetected	Very low

## 4 Discussion

This systematic review demonstrated that fluorescent adhesives significantly improve debonding efficiency by reducing residual adhesive and removal time, without compromising bond strength.

The reduction in residual adhesive corroborates previous findings demonstrating enhanced visualization with fluorescence‐aided techniques (Stadler et al. 2019; Engeler et al. 2021), further supported by studies investigating ultraviolet‐assisted detection and removal methods (Magni et al. 2022; Lai et al. 2019; Bush et al. 2010; Albertini et al. 2020; Ozcan et al. 2008).

No significant differences in SBS align with previous reports (Namura et al. 2010; Rossato et al. 2020), suggesting that fluorescent additives do not adversely affect mechanical properties. Similarly, EDI results indicate that enamel damage is primarily influenced by operator technique rather than adhesive composition.

### 4.1 Comparison With Existing Literature

The lack of significant SBS differences aligns with Rossato et al. (2020), who reported comparable SBS between fluorescent (Orthocem UV Trace, 12.69 MPa) and conventional adhesives (Transbond XT, 13.80 MPa), and Namura et al. (2010), where fluorescent dye up to 0.002% maintained clinically acceptable SBS. Conversely, AlSamak et al. (2023) and Asokan et al. (2024) found higher SBS with fluorescent adhesives, possibly due to chemical composition or curing variations (e.g., UV light at 395 nm in Asokan et al. (2024)). Reynolds (1975) suggested an optimal SBS of 6–8 MPa for clinical efficacy, a threshold exceeded by all adhesives in our meta‐analysis, supporting their adequacy despite the use of fluorescent additives. The significant reduction in the residual adhesive area corroborates Engeler et al. (2021) and Stadler et al. (2019), who noted enhanced visualization with fluorescence‐aided techniques (FIT), reducing remnants via UV detection. Similarly, Ribeiro et al. (2017) reported lower ARI scores (0.25 vs. 0.80 mm^2^) with UV light, reflecting easier remnant identification. The faster removal time aligns with that reported by Farronato et al. (2021) (38 s vs. 77 s) and Moecke et al. (2022), which is attributed to the improved visual contrast under UV. However, EDI findings contrast with Kaneshima et al. (2018), where UV increased enamel marks (*p* = 0.01), suggesting operator‐driven damage not captured in our smaller EDI meta‐analysis (*n* = 3). Recent studies have further highlighted the importance of thermocycling when evaluating post‐debonding enamel integrity. Qali et al. (2024) demonstrated that different curing techniques combined with thermocycling significantly influence enamel micro‐crack formation, debonding, and failure modes of ceramic brackets. Similarly, Alsulaimani et al. (2024) reported that thermocycling exacerbates enamel microcracks after metal bracket debonding. These findings align with our observation that EDI showed no difference between fluorescent and conventional adhesives and underscore that any potential micro‐crack risk is more related to thermal stresses and debonding mechanics than to the presence of fluorescent additives themselves.

The primary clinical rationale for fluorescent adhesives is enhanced visibility of adhesive remnants. However, the included studies varied considerably in experimental conditions (e.g., adhesive composition, curing protocols, UV wavelength, tooth type, and operator experience). This variability, particularly for bond‐strength outcomes, is reflected in the high heterogeneity observed and limits direct comparability. We therefore interpreted bond‐strength and ARI results cautiously, prioritizing visibility‐related outcomes (residual adhesive area and removal time) as more aligned with the intended mechanism of fluorescent technology.

### 4.2 Interpretation of Findings

Fluorescent adhesives leverage UV or photochromic properties to enhance visibility of tooth‐colored composites against enamel, thereby allowing clinicians to distinguish adhesive remnants more accurately and reduce both residual adhesive area and removal time. This result was fully in line with our expectations and the underlying mechanism of FIT (Stadler et al. 2019; Schott and Meller 2018). Although residual adhesive area is fundamentally an adhesive characteristic, its significant reduction in the fluorescent group is most plausibly explained by improved visibility rather than inherent differences in adhesive mechanical properties.

The non‐significant SBS and ARI differences may reflect fluorescence additives (e.g., coumarin dye (Namura et al. 2010); photochromic dye (Yan et al. 2024)) altering the adhesive structure without compromising bond strength, although high heterogeneity (*I* ^2^ = 93.07% for SBS) suggests variability in adhesive formulations, curing protocols (e.g., wavelength and duration), and tooth types. The EDI consistency (*I* ^2^ = 0%) indicates that fluorescence does not inherently protect enamel, aligning with Yan et al.'s (2024) observation that no tool completely eliminates enamel damage during removal. Sensitivity analysis revealed a higher ARI in human teeth (*p* = 0.02) post‐bovine exclusion, possibly due to enamel color similarity with composites, enhancing remnant detection in fluorescent groups, unlike SBS (*p* = 0.22), where structural differences may be less influential.

#### 4.2.1 Exploration of Heterogeneity

Substantial to very high heterogeneity was observed across several outcomes (*I* ^2^ = 93.07% for SBS, *I* ^2^ = 99.86% for residual adhesive surface area, and *I* ^2^ = 99.77% for removal time). To explore sources of heterogeneity, pre‐planned subgroup analyses by tooth type (bovine vs*.* human teeth) were conducted. These sensitivity analyses showed that exclusion of bovine samples did not significantly alter the SBS result (*p* = 0.22) but resulted in a statistically significant increase in ARI scores for fluorescent adhesives (*p* = 0.02). Other major sources of heterogeneity likely include variations in adhesive formulations and fluorescent additives, differences in illumination protocols (UV/FIT vs. conventional light), operator experience, and methods of outcome measurement. Formal subgroup meta‐analysis according to illumination method was not performed because the limited number of studies per outcome (3–6) and confounding between adhesive type and illumination technique did not allow reliable estimation. Therefore, pooled estimates for outcomes with extremely high heterogeneity should be interpreted cautiously.

### 4.3 Clinical Implications

These findings suggest that fluorescent adhesives offer practical advantages in orthodontic debonding, reducing cleanup time and residual adhesive without compromising bond strength, as evidenced by SBS exceeding the Reynolds (1975) threshold and clinical failure rates (2.5% vs. 5%, Rossato et al. (2020)) within acceptable limits (< 10%, Cal‐Neto and Miguel (2005)). Enhanced visualization could minimize enamel abrasion by limiting over‐instrumentation, although EDI data suggest that this benefit is operator dependent. Clinicians might adopt FIT‐ or UV‐assisted protocols, particularly for buccal surfaces (Engeler et al. 2021), but cost‐benefit analyses are needed given the higher expense of fluorescent adhesives and their limited enamel protection. Narratively, fluorescence intensity (Chung et al. 2024; Wu et al. 2023) and color difference (Kim et al. 2018) improved remnant detection down to 50 µm, supporting the use of QLF or FIT in precise cleanup.

### 4.4 Strength of Evidence and Clinical Translation

The majority of the included studies (17 out of 19) were in vitro investigations, with only two in vivo studies available. One of the in vivo studies involved only a single patient (Albertini et al. 2022). Although in vitro studies provide valuable controlled data on visibility, bond strength, and cleanup efficiency, they cannot fully replicate the complexities of the clinical environment, including saliva, occlusion, patient factors, and long‐term enamel responses. Therefore, the observed advantages in residual adhesive reduction and removal time should be interpreted with caution. Clinical evidence remains insufficient to support routine adoption of fluorescent adhesives in daily orthodontic practice.

### 4.5 Strengths and Limitations

The strengths of this review include its comprehensive scope (19 studies, 2010–2024), evaluation of multiple outcomes (SBS, ARI, EDI, etc.), and use of rigorous meta‐analysis with subgroup analyses (cow vs. human teeth). The inclusion of in vitro and in vivo data has broadened the relevance of this study. However, these limitations should be considered when interpreting the findings of this study. Most evidence was laboratory‐based (17/19 studies), which limits clinical generalizability. High heterogeneity (e.g., *I* ^2^ = 99.77% for removal time) reflects variability in adhesive types, operator skill, and measurement methods (e.g., stereomicroscopy vs. SEM), which reduces precision. The moderate quality of the included studies (CRIS: mostly moderate risk) due to absent blinding and randomization, alongside small in vivo samples (e.g., Albertini et al. (2022): 1 patient) further constrains confidence. The narrative synthesis of eight studies highlights diverse outcomes (e.g., cytocompatibility and bond failure), but insufficient data precluded the meta‐analysis.

### 4.6 Recommendations for Future Research

To address these gaps, future studies should prioritize standardized protocols for adhesive application, curing (e.g., consistent wavelength and distance), and removal (e.g., burr type and polishing) to reduce heterogeneity. Larger, randomized clinical trials with long‐term follow‐up are needed to assess enamel preservation and bond durability, building on Rossato et al.'s (2020) 2‐year data. Investigating the optimal fluorescence concentrations (e.g., 0.002% dye (Namura et al. 2010); 5% photochromic (Yan et al. 2024)) and their cost‐effectiveness could guide material development. Exploring operator training effects on EDI and removal time, as suggested by Kaneshima et al. (2018), would clarify the technique dependency.

## 5 Conclusion

This systematic review and meta‐analysis of 19 studies, predominantly in vitro (17/19), demonstrated that fluorescent orthodontic adhesives significantly reduced the residual adhesive surface area (MD −0.57 mm^2^, *p* = 0.04) and removal time (MD −24.89 s, *p* = 0.05) compared to conventional adhesives, without notable differences in SBS (MD 1.07 MPa, *p* = 0.61), ARI (MD 0.18, *p* = 0.20), or EDI (MD −0.04, *p* = 0.73). Enhanced visualization via UV or FIT techniques aids in efficient cleanup, although enamel protection depends on operator skills. Given the predominance of laboratory‐based evidence, limited clinical data, and high heterogeneity, these findings should be interpreted cautiously. Well‐designed randomized clinical trials with adequate sample sizes are essential to validate these results before widespread clinical implementation.

## Author Contributions

**Maryam Omidkhoda:** conceptualization, methodology, writing – review and editing, supervision. **Erfan Bardideh:** data curation, formal analysis. **Alireza Nowabadi:** investigation, data extraction. **Mostafa Entezari:** conceptualization, methodology, formal analysis, writing – original draft, writing – review and editing, supervision, project administration.

## Conflicts of Interest

The authors declare no conflicts of interest.

## Data Availability

The data that support the findings of this study are available from the corresponding author upon reasonable request.
